# Ethyl 1-(2-hy­droxy­eth­yl)-2-propyl-1*H*-benzimidazole-5-carboxyl­ate

**DOI:** 10.1107/S1600536811037421

**Published:** 2011-09-30

**Authors:** Nurasyikin Hamzah, Nurziana Ngah, Shafida Abd Hamid, Aisyah Saad Abdul Rahim

**Affiliations:** a Kulliyyah of Science, International Islamic University Malaysia, Bandar Indera Mahkota, 25200 Kuantan Pahang, Malaysia; b School of Pharmaceutical Sciences, Universiti Sains Malaysia, 11800 Penang, Malaysia

## Abstract

In the title compound, C_15_H_20_N_2_O_3_, the benzimidazole ring is essentially planar, with a maximum deviation from the mean plane of 0.012 (1) Å. The crystal structure is stabilized by inter­molecular O—H⋯N hydrogen bonds, forming centrosymmetric dimers, which are connected in the [100] direction through weak C—H⋯O contacts.

## Related literature

For the synthesis of the title compound, see: Arumugam *et al.* (2010 ▶); Kappe (2004 ▶). For general background and therapeutic properties of benzimidazole derivatives, see: Rao *et al.* (2002 ▶); Khalafi-Nezhad *et al.* (2005 ▶); Tonelli *et al.* (2010 ▶); Chen *et al.* (2007 ▶). For the low-temperature device used in the data collection, see: Cosier & Glazer (1986 ▶).
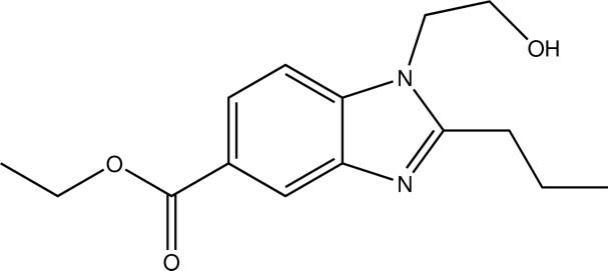


## Experimental

### 

#### Crystal data

C_15_H_20_N_2_O_3_
*M* *_r_* = 276.33
Triclinic, 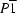

*a* = 8.5081 (3) Å
*b* = 8.5573 (3) Å
*c* = 10.0117 (4) Å
α = 94.671 (3)°
β = 106.903 (2)°
γ = 98.334 (3)°
*V* = 684.16 (4) Å^3^
*Z* = 2
Mo *K*α radiation
μ = 0.09 mm^−1^
*T* = 100 K
0.60 × 0.20 × 0.07 mm

#### Data collection

Bruker SMART APEXII CCD area-detector diffractometer
Absorption correction: multi-scan (*SADABS*; Bruker, 2009 ▶) *T*_min_ = 0.946, *T*_max_ = 0.994
10526 measured reflections
2401 independent reflections
2075 reflections with *I* > 2σ(*I*)
*R*_int_ = 0.026

#### Refinement

*R*[*F* ^2^ > 2σ(*F* ^2^)] = 0.036
*wR*(*F* ^2^) = 0.087
*S* = 1.08
2401 reflections
187 parameters
H atoms treated by a mixture of independent and constrained refinement
Δρ_max_ = 0.20 e Å^−3^
Δρ_min_ = −0.25 e Å^−3^

### 

Data collection: *APEX2* (Bruker, 2009 ▶); cell refinement: *SAINT* (Bruker, 2009 ▶); data reduction: *SAINT*; program(s) used to solve structure: *SHELXTL* (Sheldrick, 2008 ▶); program(s) used to refine structure: *SHELXTL*; molecular graphics: *SHELXTL*; software used to prepare material for publication: *SHELXTL* and *PLATON* (Spek, 2009 ▶).

## Supplementary Material

Crystal structure: contains datablock(s) global, I. DOI: 10.1107/S1600536811037421/bh2376sup1.cif

Structure factors: contains datablock(s) I. DOI: 10.1107/S1600536811037421/bh2376Isup2.hkl

Supplementary material file. DOI: 10.1107/S1600536811037421/bh2376Isup3.cml

Additional supplementary materials:  crystallographic information; 3D view; checkCIF report

## Figures and Tables

**Table 1:** Hydrogen-bond geometry (Å, °)

*D*—H⋯*A*	*D*—H	H⋯*A*	*D*⋯*A*	*D*—H⋯*A*
O3—H3*A*⋯N2^i^	0.86 (3)	1.98 (2)	2.8047 (17)	159.6 (17)
C11—H11*A*⋯O2^ii^	0.99	2.48	3.2901 (19)	139

Symmetry codes: (i) 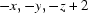; (ii) 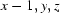.

**Fig. 1.**
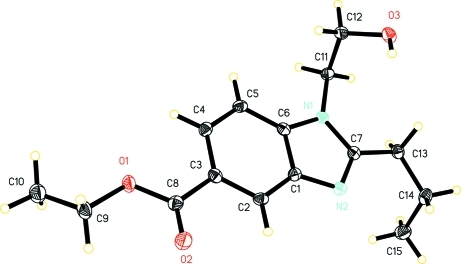
The molecular structure of the title molecule,with displacement ellipsoids drawn at the 50% probability level.

**Fig. 2.**
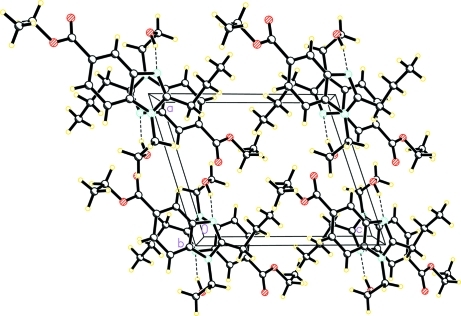
The molecular packing of the title molecule viewed down the b-axis.

## supplementary crystallographic information

### Comment

Benzimidazole compounds possess diverse functions in biological activities such as anti-HIV (Rao *et al.*, 2002), antibacterial (Khalafi-Nezhad *et al.*, 2005), antiviral (Tonelli *et al.*, 2010) and antifungal (Chen *et al.*, 2007). On the other hand, the use of microwave irradiation to assist the chemical process helps to reduce the reaction time, producing better yields and cleaner reactions (Kappe, 2004). In continuation of our study on benzimidazole derivatives (Arumugam *et al.*, 2010), we report herein the crystal structure of the title compound.

In the title molecule (Fig. 1), the benzimidazole ring [C1···C6/N1/C7/N2] is essentially planar with maximum deviation of 0.012 (1) Å for atom C4. The bond lengths and angles are in normal ranges and are in agreement with those of ethyl 1-*sec*-butyl-2-phenyl-1*H*- benzimidazole-5-carboxylate (Arumugam *et al.*, 2010). In the crystal structure, the molecule is stabilized by O3—H3A···N2 intermolecular hydrogen bond (symmetry code as in Table 1) to form dimers, which are further connected *via* weak C—H···O contacts to give chains in the [100] direction (Fig. 2).

### Experimental

A mixture of ethyl 3-amino-4-[(2-hydroxyethyl)-amino]benzoate (0.10 g, 0.22 mmol), K10-montmorillonite (0.26 g), butyraldehyde (0.07 g, 0.95 mmol) and 1 ml of MeCN were irradiated in CE*M*^TM^ microwave at 80 °C, 150 W, 5 bar and hold for 5 minutes. Then, another aliquot of aldehyde was added and the reaction was irradiated again at the same conditions as before. The progress of the reaction was monitored by TLC (Hex:EtOAc, 1:4). After completion, the mixture was filtered, washed with DCM and evaporated *in vacuo*. The resulting crude mixture was chromatographed with PLC (Hex:EtOAc,1:4). The desired compound was recrystallized with hot EtOAc which was slowly evaporated to give colorless single crystals.

### Refinement

X-ray data were collected at low temperature (Cosier & Glazer, 1986). The hydroxyl H atom was located in a difference map and refined freely. The remaining H atoms attached to C atoms were fixed geometrically and refined using the riding model, with C—H = 0.95–0.99 Å and with *U*_iso_(H)=1.2 or 1.5*U*_eq_(C). A rotating group model was applied to the methyl groups.

### Crystal data

**Table:**

C_15_H_20_N_2_O_3_	*Z* = 2
*M**_r_* = 276.33	*F*(000) = 296
Triclinic, *P*1	*D*_x_ = 1.341 Mg m^−^^3^
Hall symbol: -P 1	Mo *K*α radiation, λ = 0.71073 Å
*a* = 8.5081 (3) Å	Cell parameters from 5332 reflections
*b* = 8.5573 (3) Å	θ = 2.1–25.0°
*c* = 10.0117 (4) Å	µ = 0.09 mm^−^^1^
α = 94.671 (3)°	*T* = 100 K
β = 106.903 (2)°	Plate, colourless
γ = 98.334 (3)°	0.60 × 0.20 × 0.07 mm
*V* = 684.16 (4) Å^3^	

### Data collection

**Table:**

Bruker SMART APEXII CCD area-detector diffractometer	2401 independent reflections
Radiation source: fine-focus sealed tube	2075 reflections with *I* > 2σ(*I*)
graphite	*R*_int_ = 0.026
Detector resolution: 83.66 pixels mm^-1^	θ_max_ = 25.0°, θ_min_ = 2.1°
φ and ω scans	*h* = −10→10
Absorption correction: multi-scan (*SADABS*; Bruker, 2009)	*k* = −10→10
*T*_min_ = 0.946, *T*_max_ = 0.994	*l* = −11→11
10526 measured reflections	

### Refinement

**Table:**

Refinement on *F*^2^	Primary atom site location: structure-invariant direct methods
Least-squares matrix: full	Secondary atom site location: difference Fourier map
*R*[*F*^2^ > 2σ(*F*^2^)] = 0.036	Hydrogen site location: inferred from neighbouring sites
*wR*(*F*^2^) = 0.087	H atoms treated by a mixture of independent and constrained refinement
*S* = 1.08	*w* = 1/[σ^2^(*F*_o_^2^) + (0.0378*P*)^2^ + 0.2619*P*] where *P* = (*F*_o_^2^ + 2*F*_c_^2^)/3
2401 reflections	(Δ/σ)_max_ < 0.001
187 parameters	Δρ_max_ = 0.20 e Å^−^^3^
0 restraints	Δρ_min_ = −0.25 e Å^−^^3^
0 constraints	

### Special details

**Table:**

Experimental. The crystal was placed in the cold stream of an Oxford Cryosystems Cobra open flow nitrogen cryostat (Cosier & Glazer, 1986) operating at 100.0 (1) K.

### Fractional atomic coordinates and isotropic or equivalent isotropic displacement parameters (Å^2^)

**Table:**

	*x*	*y*	*z*	*U*_iso_*/*U*_eq_	
O1	0.27470 (12)	0.59246 (12)	0.65294 (11)	0.0183 (3)	
O2	0.45117 (13)	0.43517 (13)	0.75699 (12)	0.0241 (3)	
O3	−0.40580 (13)	−0.06135 (12)	0.88681 (11)	0.0184 (3)	
H3A	−0.315 (3)	−0.088 (2)	0.879 (2)	0.045 (6)*	
N1	−0.14250 (14)	0.23821 (13)	0.99931 (12)	0.0132 (3)	
N2	0.12077 (14)	0.19893 (14)	1.09005 (12)	0.0138 (3)	
C1	0.11012 (17)	0.28238 (16)	0.97476 (15)	0.0134 (3)	
C2	0.23274 (18)	0.33848 (16)	0.91519 (15)	0.0145 (3)	
H2A	0.3441	0.3213	0.9524	0.017*	
C3	0.18718 (18)	0.42060 (16)	0.79936 (15)	0.0146 (3)	
C4	0.02190 (18)	0.44787 (17)	0.74524 (15)	0.0152 (3)	
H4A	−0.0053	0.5061	0.6673	0.018*	
C5	−0.10116 (18)	0.39202 (16)	0.80289 (15)	0.0149 (3)	
H5A	−0.2123	0.4100	0.7663	0.018*	
C6	−0.05419 (17)	0.30799 (16)	0.91746 (14)	0.0130 (3)	
C7	−0.03154 (17)	0.17548 (16)	1.10106 (15)	0.0134 (3)	
C8	0.31874 (18)	0.48006 (17)	0.73628 (15)	0.0160 (3)	
C9	0.39782 (19)	0.65961 (18)	0.58856 (17)	0.0207 (4)	
H9A	0.3987	0.5840	0.5086	0.025*	
H9B	0.5106	0.6813	0.6582	0.025*	
C10	0.3502 (2)	0.81088 (19)	0.53830 (17)	0.0239 (4)	
H10A	0.4265	0.8557	0.4888	0.036*	
H10B	0.3571	0.8873	0.6191	0.036*	
H10C	0.2357	0.7889	0.4741	0.036*	
C11	−0.32301 (17)	0.22441 (17)	0.97243 (15)	0.0147 (3)	
H11A	−0.3564	0.3274	0.9492	0.018*	
H11B	−0.3508	0.2007	1.0589	0.018*	
C12	−0.42069 (18)	0.09446 (17)	0.85251 (15)	0.0162 (3)	
H12A	−0.5400	0.1044	0.8252	0.019*	
H12B	−0.3809	0.1103	0.7701	0.019*	
C13	−0.08488 (18)	0.09237 (17)	1.21037 (15)	0.0158 (3)	
H13A	−0.1637	−0.0071	1.1638	0.019*	
H13B	−0.1456	0.1610	1.2540	0.019*	
C14	0.05897 (19)	0.05117 (18)	1.32650 (16)	0.0184 (3)	
H14A	0.0130	−0.0277	1.3786	0.022*	
H14B	0.1328	0.0009	1.2827	0.022*	
C15	0.1621 (2)	0.19530 (19)	1.43011 (16)	0.0238 (4)	
H15A	0.2517	0.1615	1.5019	0.036*	
H15B	0.0904	0.2445	1.4754	0.036*	
H15C	0.2107	0.2727	1.3797	0.036*	

### Atomic displacement parameters (Å^2^)

**Table:**

	*U*^11^	*U*^22^	*U*^33^	*U*^12^	*U*^13^	*U*^23^
O1	0.0174 (5)	0.0194 (5)	0.0219 (6)	0.0049 (4)	0.0097 (5)	0.0088 (5)
O2	0.0174 (6)	0.0319 (6)	0.0297 (6)	0.0102 (5)	0.0117 (5)	0.0145 (5)
O3	0.0151 (6)	0.0148 (5)	0.0271 (6)	0.0033 (4)	0.0089 (5)	0.0033 (5)
N1	0.0115 (6)	0.0135 (6)	0.0149 (6)	0.0026 (5)	0.0045 (5)	0.0019 (5)
N2	0.0145 (6)	0.0139 (6)	0.0138 (6)	0.0036 (5)	0.0051 (5)	0.0023 (5)
C1	0.0146 (7)	0.0111 (7)	0.0142 (7)	0.0036 (6)	0.0039 (6)	0.0002 (6)
C2	0.0116 (7)	0.0139 (7)	0.0170 (7)	0.0035 (6)	0.0029 (6)	−0.0004 (6)
C3	0.0154 (7)	0.0111 (7)	0.0169 (8)	0.0016 (6)	0.0054 (6)	−0.0002 (6)
C4	0.0182 (8)	0.0123 (7)	0.0141 (7)	0.0034 (6)	0.0031 (6)	0.0014 (6)
C5	0.0123 (7)	0.0142 (7)	0.0165 (7)	0.0037 (6)	0.0019 (6)	0.0001 (6)
C6	0.0141 (7)	0.0110 (7)	0.0133 (7)	0.0012 (6)	0.0046 (6)	−0.0018 (6)
C7	0.0159 (7)	0.0107 (7)	0.0137 (7)	0.0033 (6)	0.0048 (6)	−0.0008 (6)
C8	0.0176 (8)	0.0160 (7)	0.0136 (7)	0.0026 (6)	0.0037 (6)	0.0009 (6)
C9	0.0197 (8)	0.0239 (8)	0.0236 (8)	0.0036 (7)	0.0132 (7)	0.0080 (7)
C10	0.0257 (9)	0.0245 (9)	0.0221 (8)	0.0019 (7)	0.0091 (7)	0.0047 (7)
C11	0.0112 (7)	0.0164 (7)	0.0181 (8)	0.0047 (6)	0.0054 (6)	0.0034 (6)
C12	0.0127 (7)	0.0172 (8)	0.0185 (8)	0.0029 (6)	0.0040 (6)	0.0030 (6)
C13	0.0169 (8)	0.0148 (7)	0.0175 (8)	0.0027 (6)	0.0077 (6)	0.0025 (6)
C14	0.0215 (8)	0.0202 (8)	0.0176 (8)	0.0074 (6)	0.0093 (7)	0.0065 (6)
C15	0.0210 (8)	0.0295 (9)	0.0200 (8)	0.0041 (7)	0.0046 (7)	0.0048 (7)

### Geometric parameters (Å, °)

**Table:**

O1—C8	1.3471 (17)	C9—C10	1.494 (2)
O1—C9	1.4565 (18)	C9—H9A	0.9900
O2—C8	1.2094 (18)	C9—H9B	0.9900
O3—C12	1.4177 (17)	C10—H10A	0.9800
O3—H3A	0.86 (2)	C10—H10B	0.9800
N1—C7	1.3762 (18)	C10—H10C	0.9800
N1—C6	1.3785 (19)	C11—C12	1.518 (2)
N1—C11	1.4653 (18)	C11—H11A	0.9900
N2—C7	1.3202 (18)	C11—H11B	0.9900
N2—C1	1.3937 (18)	C12—H12A	0.9900
C1—C2	1.392 (2)	C12—H12B	0.9900
C1—C6	1.405 (2)	C13—C14	1.529 (2)
C2—C3	1.393 (2)	C13—H13A	0.9900
C2—H2A	0.9500	C13—H13B	0.9900
C3—C4	1.414 (2)	C14—C15	1.522 (2)
C3—C8	1.486 (2)	C14—H14A	0.9900
C4—C5	1.382 (2)	C14—H14B	0.9900
C4—H4A	0.9500	C15—H15A	0.9800
C5—C6	1.394 (2)	C15—H15B	0.9800
C5—H5A	0.9500	C15—H15C	0.9800
C7—C13	1.494 (2)		
			
C8—O1—C9	115.89 (11)	C9—C10—H10A	109.5
C12—O3—H3A	111.6 (14)	C9—C10—H10B	109.5
C7—N1—C6	106.93 (11)	H10A—C10—H10B	109.5
C7—N1—C11	127.84 (12)	C9—C10—H10C	109.5
C6—N1—C11	125.05 (12)	H10A—C10—H10C	109.5
C7—N2—C1	105.09 (11)	H10B—C10—H10C	109.5
C2—C1—N2	130.08 (13)	N1—C11—C12	111.97 (11)
C2—C1—C6	120.14 (13)	N1—C11—H11A	109.2
N2—C1—C6	109.77 (12)	C12—C11—H11A	109.2
C1—C2—C3	117.98 (13)	N1—C11—H11B	109.2
C1—C2—H2A	121.0	C12—C11—H11B	109.2
C3—C2—H2A	121.0	H11A—C11—H11B	107.9
C2—C3—C4	120.92 (13)	O3—C12—C11	113.35 (12)
C2—C3—C8	117.67 (13)	O3—C12—H12A	108.9
C4—C3—C8	121.40 (13)	C11—C12—H12A	108.9
C5—C4—C3	121.66 (13)	O3—C12—H12B	108.9
C5—C4—H4A	119.2	C11—C12—H12B	108.9
C3—C4—H4A	119.2	H12A—C12—H12B	107.7
C4—C5—C6	116.73 (13)	C7—C13—C14	114.12 (12)
C4—C5—H5A	121.6	C7—C13—H13A	108.7
C6—C5—H5A	121.6	C14—C13—H13A	108.7
N1—C6—C5	131.94 (13)	C7—C13—H13B	108.7
N1—C6—C1	105.49 (12)	C14—C13—H13B	108.7
C5—C6—C1	122.55 (13)	H13A—C13—H13B	107.6
N2—C7—N1	112.72 (12)	C15—C14—C13	113.25 (12)
N2—C7—C13	125.88 (13)	C15—C14—H14A	108.9
N1—C7—C13	121.40 (12)	C13—C14—H14A	108.9
O2—C8—O1	123.07 (13)	C15—C14—H14B	108.9
O2—C8—C3	124.77 (13)	C13—C14—H14B	108.9
O1—C8—C3	112.16 (12)	H14A—C14—H14B	107.7
O1—C9—C10	107.38 (12)	C14—C15—H15A	109.5
O1—C9—H9A	110.2	C14—C15—H15B	109.5
C10—C9—H9A	110.2	H15A—C15—H15B	109.5
O1—C9—H9B	110.2	C14—C15—H15C	109.5
C10—C9—H9B	110.2	H15A—C15—H15C	109.5
H9A—C9—H9B	108.5	H15B—C15—H15C	109.5
			
C7—N2—C1—C2	179.98 (14)	C1—N2—C7—N1	0.20 (15)
C7—N2—C1—C6	0.06 (15)	C1—N2—C7—C13	−179.22 (13)
N2—C1—C2—C3	−179.43 (14)	C6—N1—C7—N2	−0.39 (15)
C6—C1—C2—C3	0.5 (2)	C11—N1—C7—N2	174.91 (12)
C1—C2—C3—C4	0.8 (2)	C6—N1—C7—C13	179.07 (12)
C1—C2—C3—C8	−179.97 (12)	C11—N1—C7—C13	−5.6 (2)
C2—C3—C4—C5	−1.2 (2)	C9—O1—C8—O2	−0.5 (2)
C8—C3—C4—C5	179.59 (13)	C9—O1—C8—C3	178.95 (12)
C3—C4—C5—C6	0.3 (2)	C2—C3—C8—O2	16.6 (2)
C7—N1—C6—C5	−178.15 (14)	C4—C3—C8—O2	−164.22 (14)
C11—N1—C6—C5	6.4 (2)	C2—C3—C8—O1	−162.86 (12)
C7—N1—C6—C1	0.39 (14)	C4—C3—C8—O1	16.34 (19)
C11—N1—C6—C1	−175.07 (12)	C8—O1—C9—C10	−163.28 (12)
C4—C5—C6—N1	179.43 (13)	C7—N1—C11—C12	−98.59 (16)
C4—C5—C6—C1	1.1 (2)	C6—N1—C11—C12	75.92 (16)
C2—C1—C6—N1	179.78 (12)	N1—C11—C12—O3	70.35 (16)
N2—C1—C6—N1	−0.28 (15)	N2—C7—C13—C14	7.5 (2)
C2—C1—C6—C5	−1.5 (2)	N1—C7—C13—C14	−171.87 (12)
N2—C1—C6—C5	178.43 (12)	C7—C13—C14—C15	73.93 (16)

### Hydrogen-bond geometry (Å, °)

**Table:**

*D*—H···*A*	*D*—H	H···*A*	*D*···*A*	*D*—H···*A*
O3—H3A···N2^i^	0.86 (3)	1.98 (2)	2.8047 (17)	159.6 (17)
C11—H11A···O2^ii^	0.99	2.48	3.2901 (19)	139
C11—H11B···O3^iii^	0.99	2.46	3.2457 (19)	136

Symmetry codes: (i) −*x*, −*y*, −*z*+2; (ii) *x*−1, *y*, *z*; (iii) −*x*−1, −*y*, −*z*+2.
